# Multidisciplinary Diagnosis of Subcutaneous Soft Tissue Metastasis of Follicular Thyroid Carcinoma: A Case Report

**DOI:** 10.3389/fendo.2020.00235

**Published:** 2020-04-22

**Authors:** Yanfang Wang, Fang Nie, Qingqing Fang

**Affiliations:** ^1^Department of Ultrasound, Lanzhou University Second Hospital, Lanzhou University, Lanzhou, China; ^2^Department of Pathology, Lanzhou University Second Hospital, Lanzhou University, Lanzhou, China

**Keywords:** follicular thyroid carcinoma, metastasis, TERT promoter, diagnosis, case report

## Abstract

**Background:** Subcutaneous soft tissue metastasis of follicular thyroid carcinoma (FTC) is rarely diagnosed before surgery for clinicians.

**Case Report:** We present a case of a 67-year-old man with a history of FTC and papillary thyroid microcarcinoma for 5 years. Multiple protruding subcutaneous nodules of the neck were found and removed from the surface of the sternocleidomastoid muscle. Ultrasound, computed tomography and technetium-99 m pertechnetate single-photon emission computed tomography of the neck were performed before the operation, which unfortunately indicated suspicious malignant lesions. Serum Tg was > 300 ng/ml (0.83–68.0 ng/ml), TSH was 36.580 uIU/ml (0.380–4.340 uIU/ml) and AbTg was negative. The pathologic diagnosis was metastatic FTC, invading the surrounding striated muscle, adipose tissue and vessels. Immunohistochemical staining revealed the tumor cells to be positive for thyroglobulin and TTF-1. The specimens of these nodules were further investigated for TERT promoter mutation and the result revealed mutated type (position g 1,295, 228 C>T).

**Conclusion:** Preoperative diagnosis and prognostic prediction of metastatic FTC may be available through a combination of clinical, multimodal imaging and molecular genetic test (viz. multidisciplinary diagnosis). A long-term standardized follow-up is required for patients with a previous diagnosis of FTC.

## Introduction

Distant metastasis of follicular thyroid carcinoma (FTC) may occur commonly to lung, skeleton and brain, which accounted for 11–25% (1). Subcutaneous soft tissue metastasis is relatively rare and difficult to diagnose before surgery (2–4). There are few available data on subcutaneous soft tissue metastasis from FTC. The clinicopathologic features of uncommon metastases of thyroid are not well-defined, which represent potential diagnostic pitfalls and may lead to treatment uncertainties (3). Here we report a subcutaneous soft tissue metastasis from FTC to investigate the clinical multiple imaging and histopathologic and molecular genetic characteristics.

## Case Report

In Mar 2014, a 62-year-old male visited the local hospital (second-tier hospital) with a small egg-like mass on his anterior neck. According to his medical records in the local hospital, physical examination revealed a 5 × 6 cm mass on the left region of the thyroid, which is slightly hard in texture, unfixed, clearly demarcated and fully movable with swallowing. Neck ultrasonography (US) showed a 7.8 × 4.0 cm mixed mass on the left lobe of the thyroid with a central irregular liquid component and hyperechoic periphery. The mixed mass had clear boundaries and abundant flow signals in the hyperechoic area. On the right lobe, there were multiple solid and cystic nodules, and the larger one was 1.0 × 0.5 cm. No suspicious lymph node was found in the bilateral cervical lymph node region. Without further imaging examinations, the patient underwent subtotal lobectomy of the left thyroid gland at the local hospital. The pathological diagnosis was FTC and was further confirmed at the pathology department of our hospital. Half a month later, the patient was referred to our hospital for radical thyroidectomy (right total lobectomy, isthmic resection, left residual lobectomy and left central compartment lymph node dissection). Before the radical thyroidectomy, physical examination and US of the neck revealed no enlarged lymph nodes. The pathology revealed papillary thyroid microcarcinoma with surrounding nodular goiters in the right lobe, no tumor was found in the rest of the thyroid, and no metastatic lymph node was detected in the left central compartment. Soon the patient received iodine-131 (^131^I) therapy (80 mCi) at another tertiary referral hospital. The serum thyroglobulin (Tg) before the ^131^I therapy was 1.28 ng/ml (0.83–68.0 ng/ml), while TSH was 83.645 uIU/ml (0.380–4.340 uIU/ml) because of the suspension of Levothyroxine. A week after ^131^I therapy, the Tg level elevated to 2.54 ng/ml (0.83–68.0 ng/ml), but neck US showed no abnormal findings in the thyroid area and bilateral lymph node region. The ^131^I whole body scan was later performed and showed no uptake in the thyroid bed region and no abnormal uptake in the rest of the body. Since then, the patient took Levothyroxine replacement therapy and visited the local hospital for follow-up. During the follow-up period, Tg and AbTg were never checked (they should have been tested, but for some reasons they were not), and TSH raised to 48.91 uIU/ml (0.34–5.6 uIU/ml) in Jun 2015, declined to 20.84 uIU/ml in Apr 2016 and returned to normal level (3.19 uIU/ml) in Feb 2018. Neck US was performed in Dec 2017 and Feb 2018. In Dec 2017, the US showed no abnormal echo in the thyroid area after surgery. While in Feb 2018, US suggested there were several solid hypoechoic masses in the bilateral anterior neck, with the larger one (1.3 × 0.9 cm) in the right. Spot flow signals were observed within those masses. Other features were not recorded in the report. This US report may suggest disease progress, but the patient did not take any further examination and treatment measures other than taking the Levothyroxine during the following period.

**Figure 1:**
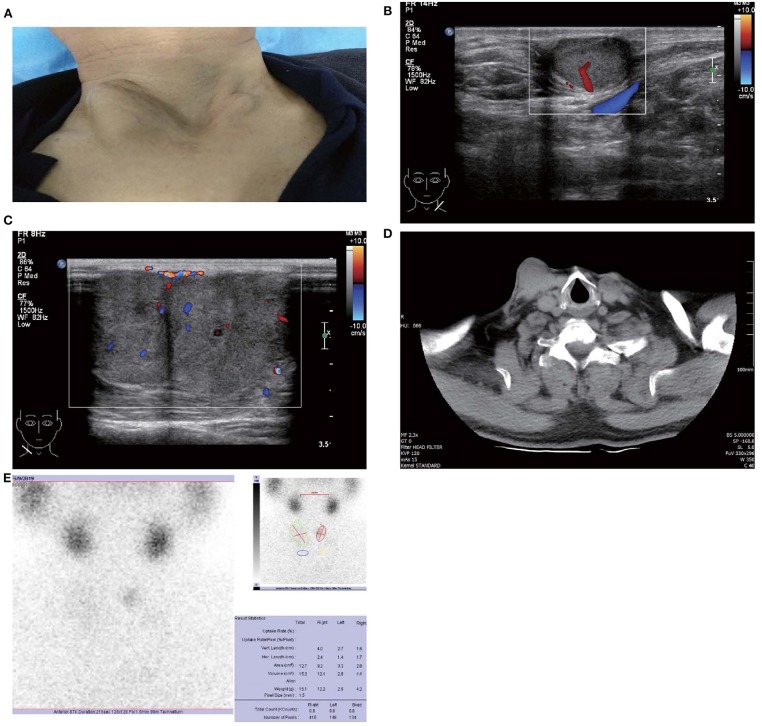
Images of patient and subcutaneous nodules. **(A)** Multiple subcutaneous soft tissue nodules of metastatic follicular thyroid carcinoma on the anterior neck. **(B)** Gray-scale ultrasound shows a hypoechoic nodule in the subcutaneous soft tissue of left neck (size were 16 × 11 mm), with wider than taller, well-defined border, irregular margin, perinodular, and intranodular flow and absence of calcification and peripheral halo. **(C)** Gray-scale ultrasound shows two solid hypoechoic nodules adjacent to each other in the subcutaneous soft tissue of right neck (size of the larger one were 28 × 23 mm), with wider than taller, partially well-defined border, partially regular margin, perinodular and intranodular flow and absence of calcification and peripheral halo. **(D)** Computed tomography shows several low-density nodules in the subcutaneous of the anterior neck. **(E)** ^99m^TcO_4_-SPECT reveals a shadow of a cold nodule in the right anterior neck region and a shadow of a nodule in the left anterior neck region.

**Figure 2:**
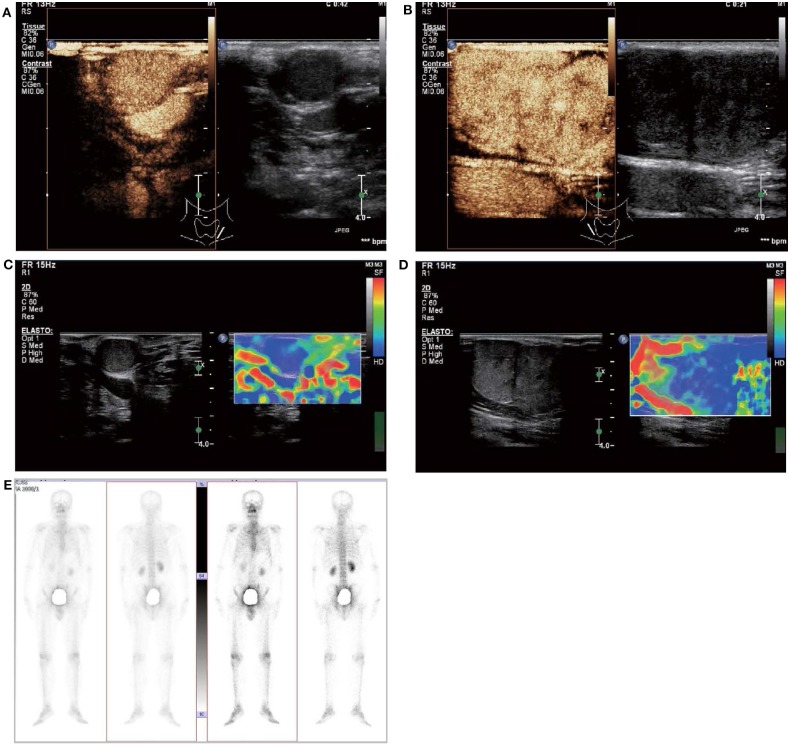
Contrast-enhanced ultrasound and elastosonography appearance of the larger subcutaneous nodules on the bilateral neck. **(A,B)** Contrast-enhanced ultrasound showed both the larger nodule in the bilateral neck are hypervascular, heterogeneous enhancement, no ring enhancement, and partially clear enhanced boundary. **(C,D)** Elastosonography displayed both the larger one were mainly composed of blue color (at least 75% of the nodule was covered in blue). **(E)** ^99m^TcO_4_-SPECT whole body scan was performed and showed no abnormal uptake in the whole body.

**Figure 3:**
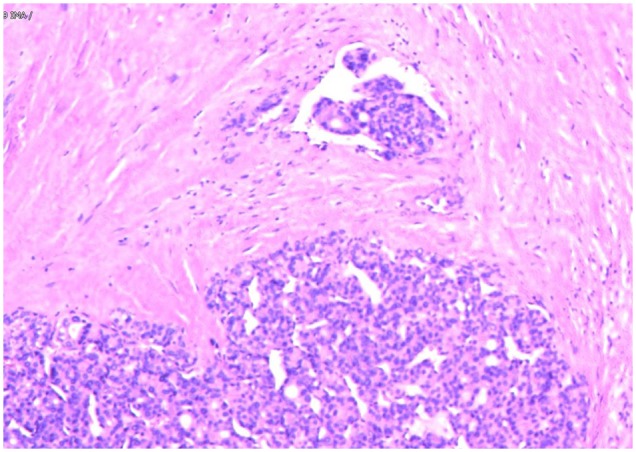
Follicular thyroid carcinoma. (Hematoxylin and eosin staining of histological slides, × 200).

**Figure 4:**
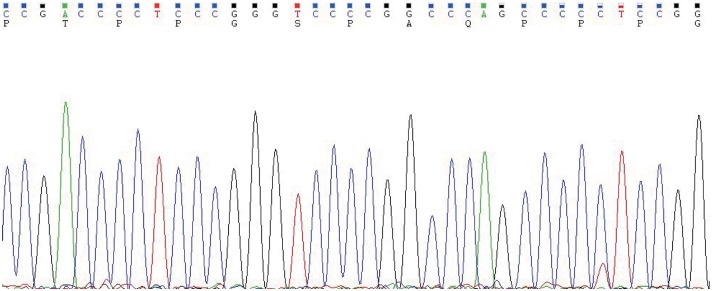
The TERT promoter testing displayed a mutation type (position g 1,295, 228 C>T). Genomic DNA was isolated from four 5 μm-thick slices of formalin-fixed paraffin-embedded metastatic follicular thyroid carcinoma samples, using a commercial DNA extraction kit (TIANamp FFPE DNA Kit, TIANGEN, Catalog No. DP130608, China), according to the instruction of the manufacturer. The TERT promoter region (2 mutation hot spots—chr5:1,295, 228 C>T and chr5:1,295, 250 C>T) was amplified with a commercial kit (TERT Genetic Mutation Detection Kit, SinoMD, Catalog No. 20090031, China), according to the manufacturer's instructions. The amplified products were sequenced with Applied Biosystems 3,500 Genetic Analyzeror and mutations were recognized on sequencing electropherograms.

In May 2019, the patient revisited our hospital with multiple asymptomatic, soft, protruding nodules on the anterior neck (Figure 1A). Thyroid function examination showed that Tg was > 300 ng/ml (0.83–68.0 ng/ml), AbTg was <15 U/ml(0-60 U/ml), TSH was 36.580 uIU/ml (0.380–4.340 uIU/ml), FT4 was 8.28 pmol/L (10.44–24.88), and T3, FT3 and TPO-Ab were within normal levels. Physical examination found several soft, unfixed, well-defined mass in the lateral thoracic bone end of the bilateral sternocleidomastoid, immovable with swallowing. The larger one was in the right, about 3 × 3 cm. Subsequently, US, computed tomography (CT) and technetium-99 m pertechnetate single-photon emission CT (^99m^TcO_4_-SPECT) of the neck were performed. Neck US revealed several solid nodules with the similar US features in the left anterior neck, the larger one displayed a well-defined border and irregular margin (Figure 1B). In the right anterior neck, two solid nodules adjacent to each other with a partially well-defined border and partial regular margin (Figure 1C) were found. All of them showed solid hypoechogenicity, wider than tall, no calcification, absent peripheral halo and perinodular and intranodular flow, while CT revealed multiple low-density nodules in the subcutaneous tissue of the bilateral anterior neck (Figure 1D). SPECT found a shadow of a cold nodule in the right anterior neck region and a shadow of a nodule in the left anterior neck region (Figure 1E). Furthermore, the contrast-enhanced ultrasound showed hyper-vascular, larger nodules of bilateral neck with heterogeneous enhancement, no ring enhancement and partially clear enhanced boundary (Figures 2A,B). Elastosonography displayed both larger nodules mainly composed of blue color (at least 75% of the nodule was covered in blue) (Figures 2C,D). The elasticity score (ES) was classified into 4 (ES 4), which correlated with malignancy (5). As the patient refused ^131^I whole body scan and 18-fluorodeoxyglucose positron-emission tomography/CT (18F-FDG-PET/CT) scan, ^99m^TcO_4_-SPECT whole body scan was performed, showing no abnormal uptake in the whole body (Figure 2E). A surgical excision was performed and these nodules were removed from the surface of the sternocleidomastoid. The histopathologic examination of these nodules was FTC, invading the surrounding striated muscle, adipose tissue and vessels. Under the microscope, the tumor cells were arranged in the form of small follicles, with acidophilic cytoplasm, enlarged, hyperchromatic nuclei and vascular invasion (Figure 3). Immunohistochemistry revealed the tumor cells to be positive for Tg and TTF-1. According to the recent AJCC cancer staging manual (6), the TNM staging of this patient was T4aN0bM0. The TERT promoter mutation testing was positive (position g 1,295, 228 C>T) (Figure 4). Preoperative and postoperative US was performed by the same imaging doctor, with the latter suggesting no residual nodules and obvious abnormal echo on the bilateral anterior neck region. Up to 6 months of thyroid function and neck US follow-up, the patient has been doing well. The latest Tg was <0.1 ng/ml (0.83–68.0 ng/ml), AbTg was <15 U/ml(0–60 U/ml), and TSH was 0.320 uIU/ml (0.380–4.340 uIU/ml). The latest neck US showed no abnormal echo in the thyroid area and bilateral anterior neck region. A long-term standardized follow-up is needed for this patient in the future.

## Discussion

FTC is the second most common differentiated thyroid malignancy. It is difficult to distinguish between thyroid follicular carcinoma and follicular adenoma before the operation. Around 5.4–11% of patients with FTC have distant metastasis, which usually has a poor prognosis and affects the survival of patients (7, 8). The most unusual metastatic site reported in the literature is the skin (3, 9). In our case, the metastatic site was the soft tissue between the skin and the surface of the sternocleidomastoid muscle. Adequate imaging follow-up and thyroid function follow-up are required despite surgical excision and ^131^I therapy.

US is the most common non-invasive imaging method preferred for thyroid nodules, which can be used in differentiating benign and malignant lesions. Although extensive experience has been accumulated in diagnosing papillary thyroid carcinoma with US, there is limited and few data available in differentiating FTC (10, 11). Previous reports have suggested that larger size, absent sonographic halo, hypoechoic appearance, absence of cystic change and tubercle-in-nodule combination with calcification favored an FTC (12, 13). In this study, the sonographic findings of the metastatic nodules were solid hypoechoic, partially clear border, irregular margin, absent calcification, absent peripheral halo, perinodular and intranodular flow, hyper-vascularity, heterogeneous enhancement, no ring enhancement, partially clear enhanced boundary and ES 4. These findings were unfortunately indicative of suspicious malignant lesions. However, there are no specific ultrasonic features for the diagnosis of FTC at present, let alone metastatic FTC. CT/MRI is more advantageous in staging but less advantageous in diagnosing than US. ^131^I- or ^99m^TcO_4_-SPECT is used to image thyroid metabolism to detect and localize abnormal thyroid tissue, especially in imaging ectopic or metastatic thyroid lesions. In the current case, ^131^I therapy after radical thyroidectomy, there was no uptake in the thyroid bed region and no abnormal uptake in the rest of the body by the ^131^I whole body scanning. It could be supposed that there was no metastatic, residual or ectopic thyroid tissue in the patient's body. But 5 years later, the ^99m^TcO_4_-SPECT revealed shadows in the bilateral anterior neck region. Linking the history and previous examinations, we considered they were metastatic lesions. However, since SPECT is a projection image from the radioactive nuclide, the image may be noisy and low in resolution. Furthermore, the lack of anatomic landmarks, as well as the possibility of physiologic or other benign uptake of this tracer, may make interpretation of images more difficult (14, 15). A further study is necessary to distinguish FTC by using image diagnostics in the future.

Immunohistochemical staining has helped confirm the tissue origin in dealing with metastatic lesions (16). From published data, immunohistochemical markers that are specific for FTC such as Tg and TTF-1 can be detected in over 95 and 100% of FTC cases (17). In our case, the Tg and TTF-1 were positive in subcutaneous metastasis nodules. Besides, the tumor cells under the microscope were arranged in the form of small follicles, with acidophilic cytoplasm, enlarged, hyperchromatic nuclei and vascular invasion. All of these findings support the diagnosis of metastatic FTC.

In thyroid cancer, TERT promoter mutations are usually presented in follicular-derived thyroid carcinoma and associated with more aggressiveness, distant metastasis, tumor recurrence and poor prognosis (18, 19). In our case, the metastasis nodules harbored TERT C228T mutation (Figure 4), which implies it may contribute to the aggressiveness of the FTC and distant metastasis. Indeed, the histopathological results showed these FTCs had invaded the surrounding striated muscle, adipose tissue and vessels. As the primary FTC was not performed in our hospital, we cannot make a molecular test for the original tumor. Therefore, we do not know whether there was a difference in molecular mutation between primary and metastatic tumors. Six months since the last surgical removal, the patient has been doing well. As the TERT mutation may indicate a poor prognosis, a long-term standardized follow-up is required for the patient.

Serum Tg is a very important laboratory indicator in clinical monitoring of recurrence and metastasis after thyroidectomy of thyroid cancer (20). Tg is a specific protein produced by the thyroid gland and secreted by thyroid follicular epithelial cells. In patients whose thyroid has been completely cleared (surgery and ^131^I therapy), there should no longer be a source of Tg in the body. If detected in the serum, it often indicates residual disease or recurrence. However, serum Tg detection is susceptible to AbTg and TSH. When AbTg is positive, it will reduce the detection value of serum Tg by chemiluminescence immunoassay, thus affecting the accuracy of disease monitoring (21). It is noteworthy that the results of different Tg detection reagents may vary greatly, so the same detection reagent should be used in the follow-up (22). Under the TSH suppression, the ability of tumor cells to secret Tg is also inhibited. To better reflect the disease status, Tg under the stimulation of TSH (TSH >30 uIU/ml) is usually preferred in clinical practice (23). And the cut-off value above 2 ng/mL following TSH stimulation is highly sensitive in identifying patients with persistent tumor (20). In this case, AbTg is negative and Tg (>300 ng/ml) was measured under the TSH stimulation (TSH: 36.580 uIU/ml). Metastasis or recurrence is highly suggestive of for this patient.

It should be noted that in this patient there could have been possible earlier progress; however, for certain reasons, it was delayed. This can be reflected by two aspects: first, during the follow-up period after ^131^I therapy, Tg and AbTg were never checked; second, in Feb 2018, the US suggested there were several solid hypoechoic masses in the bilateral anterior neck. But the patient did not take any further management. If fundamental, standardized follow-up and timely treatment measurements were administered, the patient may have smaller surgical excision areas and lighter complications. This indicates that the regular standardized follow-up and timely treatment is completely necessary for FTC patients after thyroidectomy.

In summary, subcutaneous soft tissue metastasis from FTC is unusual. Multidisciplinary diagnosis, namely, combing clinical information with multimodal imaging materials and molecular genetic test, may help in diagnosing metastatic FTC and predicting prognosis before the operation. Immunohistochemical staining of Tg and TTF-1 plays a very important role in the final histopathological diagnosis of metastatic thyroid carcinoma. Postoperatively, monitoring of circulating Tg, AbTg and TSH together with neck US is very important in the follow-up of FTC. Since rare metastases identification could have a significant impact on patient management (3), the necessity for long-term standardized follow-up should be fully informed to patients, and the surgeons and physicians should take the metastasis into consideration in patients with a history of FTC.

## Data Availability Statement

The datasets generated for this study can be found in the article.

## Ethics Statement

Written informed consent was obtained from the individual for the publication of any potentially identifiable images or data included in this article.

## Author Contributions

FN: case review. YW: clinical data, imaging data, design, analysis, and writing. QF: pathology data. All authors contributed to the manuscript work and approved the submitted version.

## Conflict of Interest

The authors declare that the research was conducted in the absence of any commercial or financial relationships that could be construed as a potential conflict of interest.
